# The Nose as a Route for Therapy: Part 1. Pharmacotherapy

**DOI:** 10.3389/falgy.2021.638136

**Published:** 2021-02-22

**Authors:** Cemal Cingi, Nuray Bayar Muluk, Dimitrios I. Mitsias, Nikolaos G. Papadopoulos, Ludger Klimek, Anu Laulajainen-Hongisto, Maija Hytönen, Sanna Katriina Toppila-Salmi, Glenis Kathleen Scadding

**Affiliations:** ^1^Department of Otolaryngology, Eskişehir Osmangazi University, Eskişehir, Turkey; ^2^Department of Otolaryngology, Kirikkale University, Kirikkale, Turkey; ^3^Allergy Department, 2nd Pediatric Clinic, National and Kapodistrian University of Athens, Athens, Greece; ^4^Royal Manchester Children's Hospital, Manchester, United Kingdom; ^5^Centre for Rhinology and Allergology, Wiesbaden, Germany; ^6^Department of Otorhinolaryngology-Head and Neck Surgery, Helsinki University Hospital, Helsinki, Finland; ^7^Skin and Allergy Hospital, Helsinki University Central Hospital, Helsinki, Finland; ^8^Faculty of Medicine, The Haartman Institute, University of Helsinki, Helsinki, Finland; ^9^University College London Hospitals NHS Foundation Trust, London, United Kingdom; ^10^Royal National Throat Nose and Ear Hospital, London, United Kingdom

**Keywords:** intranasal route, nasal epithelium, mucociliary clearance, allergic rhinitis, chronic rhinosinusitis, lysine aspirin, saline douche, drug delivery

## Abstract

This article reviews nasal structure and function in the light of intranasal pharmacotherapy. The nose provides an accessible, fast route for local treatment of nose and sinus diseases, with lower doses than are necessary systemically and few adverse effects. It can also be used for other medications as it has sufficient surface area protected from local damage by mucociliary clearance, absence of digestive enzymes, responsive blood flow, and provides a rapid route to the central nervous system.

## Introduction

Medicines are usually given orally or systemically by injection: intramuscular or intravenous. Indeed when patients are asked about their drug history the use of inhalers or sprays is often inadvertently omitted, unless specifically requested. However, other routes not only exist, but can prove more effective in placing a drug accurately, often using smaller doses. One such is the intranasal route, now brought to prominence by SARS-CoV2, which uses it to invade the body.

The nose, even though obvious, “it's as plain as the nose on your face” is an English expression, is often disregarded by non-otorhinolaryngologists. However, it has much to recommend it: as an organ for conditioning inspired air, for immune defense, for hosting smell receptors and for application of therapy. The leading role of the epithelium in respiratory diseases such as Allergic Rhinitis (AR) and Chronic Rhinosinusitis with Nasal Polyps (CRSwNPs) has become apparent in recent years and the ability to interact with it by direct application of molecules, rather than allowing them to reach it via the circulation, having been absorbed via the gut or injected into the system, seems sensible.

Part 1 of this review article involves nasal pharmacotherapy. It begins with a consideration of nasal structure and function, including the nature of the pseudostratified columnar ciliated respiratory epithelium. It is important to understand nasal anatomy, histology, innervation, and blood supply in order to assess the nasal cavity as a route for a particular drug. Necessary factors are a large surface area for absorption and high blood flow for transport. Factors which might interfere with drug absorption are vasoconstriction secondary to stimulation of the adrenergic nerves or irritation stimulating the 5th nerve and causing the 7th to respond by increased glandular mucus secretion, washing away the therapeutic product into the nasopharynx, where it is swallowed. Nasal pH and the lipophilicity of a drug are also relevant.

The article continues with various intranasal therapies, varying from those used locally to treat respiratory diseases, to those countering entirely other problems, such as diabetes insipidus. Cheap and simple measures such as nasal saline or lysine aspirin can prove prophylactic, therapeutic or both. Unfortunately the use of nasal adrenalin for rescue in anaphylaxis was considered too commercially sensitive for inclusion in this paper.

Prevention of COVID-19 infection by copper—containing face masks has just emerged as an idea, doubtless other intranasal approaches will follow. The nose, overlooked for so long, is finally becoming prominent.

Part 2 will follow with a consideration of nasal immunology and immunologically—based nasal therapeutics.

## Nasal Structure and Function

### Nasal Anatomy

The nasal cavity is a midline airway passage of some 15 ml in volume and 14 cm in length in the adult, extending from the nares anteriorly to the post- nasal space. Approximately cylindrical, it is divided by the nasal septum into two nostrils. Above it are sinuses: frontal, ethmoid, and sphenoid, from front to back; maxillary sinuses are present on each side. Its surface area is ~160 cm^2^, but if microvilli are included this rises to nearly 10 m.^2^

### Nasal Histology

The vestibular entrance to the nose is lined internally by a squamous epithelial layer. The lining changes to a pseudostratified columnar epithelium of respiratory type, bearing cilia and with numerous glands of serous and mucinous type, after the first 1–2 cm (1). This lines most of the respiratory tract, including the sinuses, down to the alveoli.

### Epithelial Cells (Cilia)

The epithelium functions as a physical block on the entry of pathogens into the deeper tissues. The movement of their cilia occurs in the deeper sol layer of the nasal mucus, with a stiff- armed forward stroke followed by a limp backward one. This pushes the mucus, including the upper gel layer into which particles entering the nose become contained, backwards in the direction of the nasopharynx. These substances are then usually swallowed. This phenomenon is termed mucociliary clearance (2). Intranasal drugs therefore have a short absorption window before being cleared to the throat and swallowed. This clearance mechanism means that corticosteroids applied locally do not cause atrophy, unlike dermal application, provided septal deposition is avoided. Unlike the oral cavity and gut lumen there is no regular secretion of digestive enzymes capable of disrupting peptides into the nasal cavity, though peptidases may be released from epithelial cells upon stimulation by allergen.

The epithelium also plays a role in regulating inflammation by the secretion of cytokines (3).

**Figure 1:**
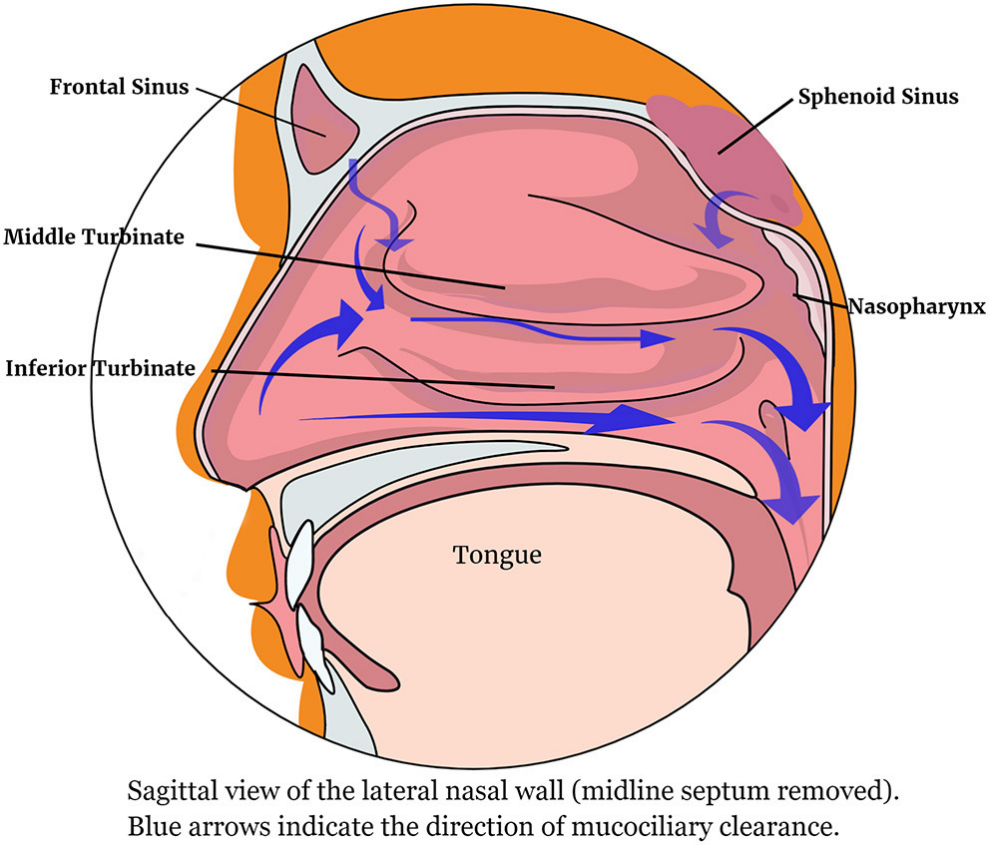
The lateral nasal wall showing the direction of mucociliary clearance.

Figure 1 shows the lateral nasal wall and the direction of mucus movement.

### Endothelium (Sub-epithelial Blood Vessels)

The nasal lining has an abundant vascular supply via capillaries lined by endothelial cells. The endothelial layer is of minimal thickness, so that heat can be rapidly transferred to inhaled air. Encircling the endothelium is a smooth muscle layer, which acts to narrow or widen the vasculature. This regulatory action on vessel diameters is a key feature of inflammation (3).

### Mucous Glands

Small serous glands, similar to salivary glands are scattered in the front part of the nose. They secrete watery fluid, sometimes visible as droplets in cold conditions.

Seromucous glands secreting more proteineous secretions are located in the lamina propria elsewhere in the nasal cavity. They deposit mucus onto the external surface of the epithelium. The secreted mucus can immobilize external matter and helps to conserve the integrity of the physical barrier. Parasympathetic nervous impulses result in more mucus being synthesized and excreted. The mucus contains lysozyme and immunoglobulin A, which help to attack potentially invasive microbial organisms (3, 4).

### Physiology of the Nose

The nasal cavity has a variety of roles, notably respiratory, olfactory, immunological, and the conditioning of air before entry into the lower respiratory tract. The cavity offers a very extensive, humid surface area which is optimal for adjusting the temperature and humidity of inhaled air prior to its passage toward the oxygen exchanging pulmonary surfaces. Mucus secreted by the nasal lining stops external matter from damaging the epithelial layer, especially in the course of an inflammatory response. The nose is the only human organ where olfaction occurs and depends on specialized sensory neurones that form part of the olfactory nerve.

### Nasal Cycle

There is a continuously operating nasal cycle whereby the two sides of the cavity alternate between congestion and decongestion (3). In adults without health problems, the total resistance to airflow offered by the nose remains fairly constant, although there is an alternating pattern of one side of the nasal interior offering a greater level of resistance to airflow, whilst the other remains fully patent, followed by the inverse (5, 6). This pattern of flow restriction is referred to as the nasal cycle. It is produced by alternating changes in blood flow to the turbinates and the tubercle of the septum. In healthy individuals, this cycle occurs without the person noticing it, since there is no net alteration in how much airflow through the nose can take place. Likewise, the moisture content of inhaled air passing to the lungs does not vary (7). The hypothalamus contains the pacemaker area controlling the nasal cycle (8).

### Vascular and Lymphatic Supply

The blood supply to the nasal cavity is extensive, involving six arterial branches, forming a good route for drug administration. There are two main sources of vascular supply to the nose: the internal and external carotid arteries. The former gives rise to the ophthalmic artery and its branches, the anterior ethmoid artery and the posterior ethmoid artery. From the latter arise the sphenopalatine, greater palatine, superior labial, and angular arteries.

The posterior and inferior portions of the interior aspect of the lateral nasal wall receive arterial blood from the sphenopalatine artery, whilst its superior portion is supplied by the ethmoid arteries, both anterior and superior. The septum of the nose receives a vascular supply from these same three arteries. This supply is augmented in the anterior portion by the superior labial artery and in the posterior portion the greater palatine artery makes its contribution. Little's area (also referred to as the Kiesselbach plexus) is an area situated in the most anterior and inferior third of the septum and is where most epistaxes occur. Here the principal arteries providing vascular supply to the nose all anastomose.

The nasal venous network has a similar layout to that of the arteries. The arterial blood flow into the nasal region is overabsorbed into the nasal veins, with the excess draining into the lymphatics, forming a good route for vaccine delivery. The veins do not possess valves and thus communicate directly with the cavernous sinus. In this way, they may render it easy for pathogens and drugs to disseminate within the cranium. Although the nose enjoys a rich vascular supply, smokers suffer from impaired recovery following surgery to the nose.

The lymph vessels originate in the outer layers of the mucosa with drainage from the posterior nasal cavity toward the retropharyngeal nodes and from the anterior cavity to the superior deep cervical or submandibular nodes.

**Figure 2:**
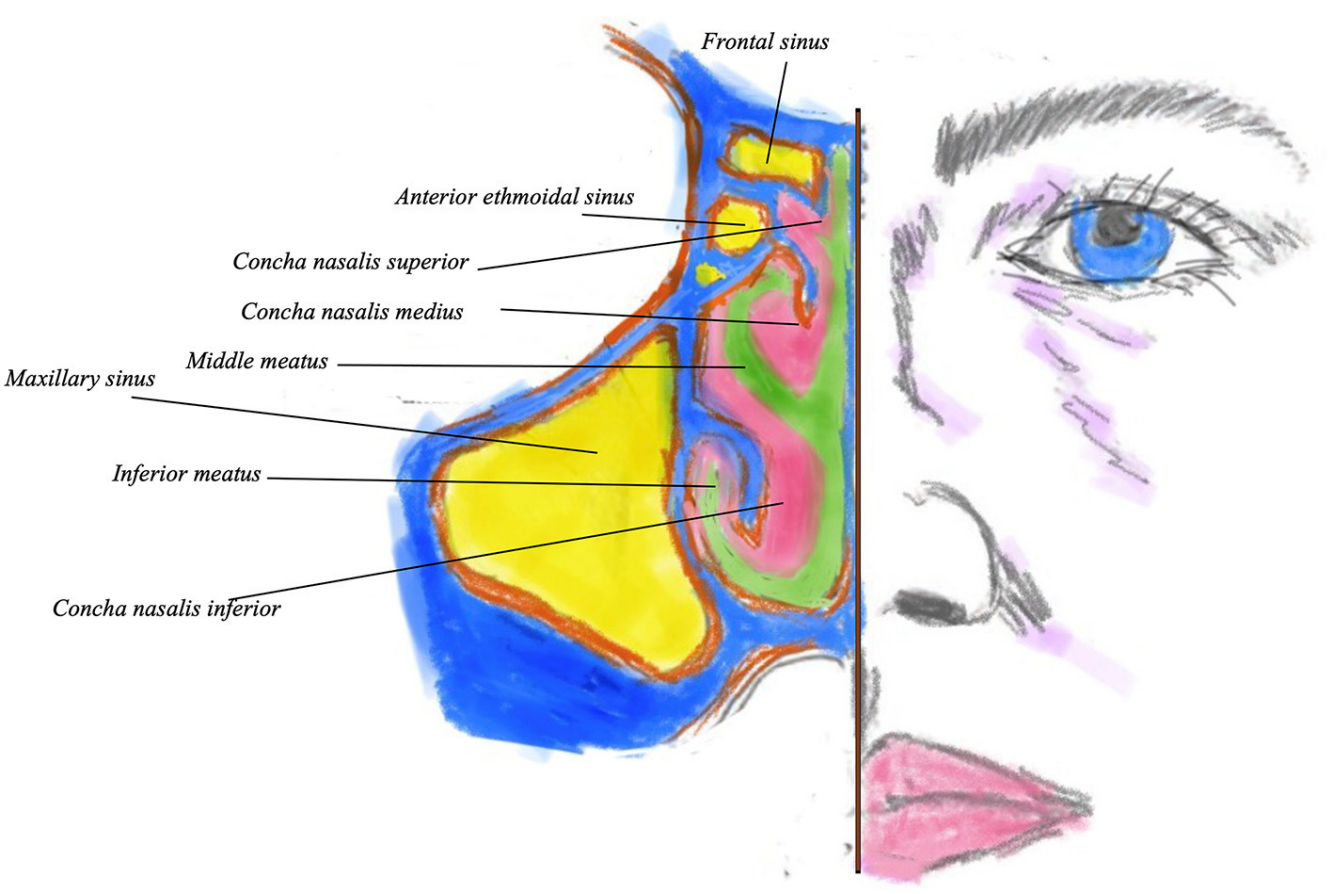
Coronal view of the nostril showing the sinuses (in yellow) turbinates and meati. Bony turbinate structure is in blue, with pink denoting the overlying mucosa. Green indicates the nasal airway.

The nasal mucosa contains a network fenestrated veins beneath the mucous membrane. These may provide some humidifying fluid. The epithelium also possesses a network of vascular erectile tissue, which is also cavernous and well-developed over the lower conchae and septum as shown in Figure 2, thus providing a good absorption route. Adrenergic vasoconstriction will decrease the rate of absorption, cholinergic vasodilatation may increase it, thus altering drug penetration.

### Nervous Supply

The extensive sensory nervous supply to the nose is provided by the initial two branches of the fifth cranial nerve (1): the ophthalmic and maxillary divisions. The latter includes the anterior superior alveolar nerve, which is important in sneezing. The 5th (trigeminal) nerve is responsible for sensing pain and irritation following nasal administration, but it is the 7th (facial) nerve which contains motor fibers and responds to such irritation by stimulating facial movements and glandular secretion.

The 1st cranial (olfactory) nerve is the only site where the central nervous system is directly expressed on the mucosal surface and is hence in contact with the external world. This gives a route for central nervous system (CNS) access for drugs, but also for pathogens.

### Parasympathetic Innervation

Parasympathetic innervation occurs via the greater superficial petrosal branch of the facial nerve. This branch combines with the deep petrosal nerve (carrying sympathetic fibers). The deep petrosal nerve emerges from the carotid plexus. Together they make up the vidian nerve within the pterygoid canal. The vidian nerve passes through the pterygopalatine ganglion, but the sympathetic fibers do not make any synaptic connections in the ganglion. Then the vidian nerve joins with fibers from the maxillary division of the fifth cranial nerve to supply the lacrimal gland, the nasal glands and the palate (1).

### Osteology

There are twin nasal bones, the superior aspects of which articulate with the frontal bone. The nasal bones articulate with the lacrimal bones on their superolateral aspect, whilst on their inferolateral aspect they articulate with the maxilla on its ascending process. In a posterior and superior direction, the osseous septum of the nose is formed by the ethmoidal perpendicular plate. The septum is thinner centrally and often bent to one side or the other (septal deviation), which may interfere with drug delivery (9). In the posterior and inferior direction there is the vomer, which contributes a portion of the choanae, leading into the nasopharynx. The bony nasal floor is formed by the premaxilla and the palate.

Situated on the lateral walls of the nasal cavity are the three conchae (superior, middle, and inferior), providing the osseous support to the turbinates, projections into the lateral wall which promote turbulent airflow, enabling particle deposition and also act as radiators, warming the inspired air. The medial wall of the maxillary sinus is situated laterally to the turbinates.

Below each turbinate there are apertures, the meatuses, named after the turbinate immediately superior to them. For example, the middle meatus into which most sinuses ventilate and drain is just below the middle turbinate. The conchae and meatus increase nasal surface area, allowing significant absorption of medications (10) (Figure 2).

Viewed from the internal aspect of the nasal cavity, the roof consists of the ethmoidal cribriform plate. Behind and below the roof and angled posteriorly lies the bony face of the sphenoid sinus (1).

### Paranasal Sinuses

These develop and enlarge after birth; it is not until some 3–7 years of age that the ethmoid and sphenoid sinuses are of significant size. The frontal sinuses develop last, not reaching full size until adolescence.

The sinuses in human beings exist as four pairs, each of which is lined by epithelial cells of the pseudostratified columnar type. The maxillary sinuses located within the maxilla and inferior to the orbit are the biggest. The frontal sinuses are within the frontal bone and are found above the orbit. The ethmoid sinuses consist of a number of separate pneumatized sacs within the ethmoid bone in between the nasal cavity and the orbit. They are divided into anterior and posterior groups, with differing drainage. The anterior ethmoids drain into the middle meatus via the ethmoid infundibulum; the posterior ethmoids sinuses drain into the superior meatus via the sphenoethmoidal recess. The sphenoid sinuses are inside the sphenoid bone (8).

It is an unresolved issue as to precisely what functions the paranasal sinuses perform, but they appear to accomplish the following (8):

They help to reduce skull weight
They allow the voice to have a more resonant quality
They help to absorb the impact of a blow to the face
They protect against abrupt changes in the temperature of the nasal cavity and thereby prevent injury to some structures that are sensitive to heat or cold
They condition air by adding moisture and warming it before it passes to the lungs
They perform an immune defensive function via the formation of nitric oxide.

## Nasal Saline

Probably the oldest and by far the most frequently used nasal treatment is that of saline. Almost all nasal morbidities [e.g. allergic rhinitis (AR), chronic rhinosinusitis (CRS), infectious rhinitis etc.] are characterized by increased nasal secretions and/or congestion and are empirically countered by patients with nasal douches. Moreover, the concept of nasal irrigation (NI) is supported by many physicians, usually as an add-on to pharmacological treatment. For diseases that follow a chronic course, and therefore need chronic treatment, concerns about drug usage are raised and non-pharmacological approaches are preferred (11, 12). This is of importance in pediatric and elderly populations where parents/caregivers are often skeptical or unwilling regarding protracted pharmacological treatments and adherence is low (13).

### Methods

There are many ways to perform NI: sprays, pumps, squeezy bottles, even plain syringes have been used, following various protocols. Moreover, the device may deliver high or low volume of saline, isotonic or hypertonic. NI are widely used and accepted, being included in therapeutic algorithms for AR and CRS (14, 15).

### Mode of Action

This, though not fully delineated, seems to be multiple. First, it humidifies and moisturizes the nasal mucosa and hypertonic saline may reduce mucosal edema. Second, it removes particles, allergens, air pollutants leading to less interaction with the mucosa and, probably, less inflammation. Third, saline seems to make the mucus thinner and more easily expelled and, in turn, mucociliary clearance is improved. Of importance, the release of inflammatory mediators such as histamine, prostaglandins and leukotrienes is reduced and/or receptors, such as ICAM-1 that are used for viral entry to the epithelium (16) are down-regulated. It seems, therefore, that apart from the “mechanical” mode of action, NI may exert immunological effects. Finally, in a post-operative setting, the removal of thick crusts, clotted blood and debris may result in faster wound healing.

### Tonicity

The first key issue that needs to be addressed is whether hypertonic solutions (i.e., > 0.9% in sodium chloride) are better than normal (iso-osmotic) saline. Such studies as are available favor the former. Mucociliary clearance is increased (17–19) and clinical studies in children with both allergic rhinitis and chronic sinusitis showed the superiority of hypertonic solutions (20–22); systematic reviews confirm these results (23, 24). In adults with CRS, the advantage of hypertonic solutions albeit probable, is less evident (25–27). Tonicities above 3% may both decrease mucociliary clearance and open tight junctions thus increasing epithelial permeability (18, 28). Post-operatively, where there is no inflammatory/allergic background, the main goal is the removal of crusts and debris. In this setting, high osmolarity seems to be of minor importance (29) whereas the volume of the NI is more crucial (30); nevertheless there are contradictory results (31).

### Saline in Allergic Rhinitis

The efficacy of NI in children and adults with AR has been well-studied. Even though these studies are characterized by large heterogeneity, they all point to increased efficacy, either as add-on to pharmacological treatment (antihistamines and/or nasal steroids) or alone, compared to no intervention at all (21, 32, 33). Indicatively, the study of 220 children (aged 5–9 years old) with AR showed the superiority of hypertonic saline (2.7%) compared to normal saline (and even more compared to no intervention) regarding nasal symptoms and turbinate swelling and/or adenoidal hypertrophy. Moreover, NI resulted in reduced antihistamine use, especially in the hypertonic NI group (21). Similarly, 44 children (5–14 years old) with seasonal AR were prescribed hypertonic NI (or not) as add on to antihistamine treatment. The active group had significantly better rhinoconjunctivitis score and less drug usage (32). Recently, 76 children and adolescents (6–18 years old) with seasonal or perennial AR, used NI with a sea-water solution supplemented with algal extracts as an add on to regular treatment. The active group showed significantly improved AR symptom control as judged by CARAT questionnaires, better combined symptom and medication scores using the MASK Allergy Diary (a mobile application designed by the ARIA group) and reduced drug usage (33). Meta-analyses also suggest that NI have no adverse events, lead to less drug usage and can be used as add-on treatment for AR (34, 35).

### Saline in Chronic Rhinosinusitis

In CRS, NI have also proven useful (22, 25, 26, 36, 37). Thirty children (3–16 years old) with rhinosinusitis were treated with either hypertonic (3.5%) or normal saline. The first group improved significantly in cough and nasal secretion/post nasal drip score, as well as radiology score, while the normal saline group showed significant improvement only in the post nasal drip score (22). Nasal patency and mucociliary clearance was studied in 80 adult patients with CRS. Both hypertonic and normal saline improved subjective symptoms (i.e., stuffiness and obstruction) and mucociliary clearance (greater effect with hypertonic saline). Nasal patency was increased with normal saline (25). Finally, a randomized control trial of 76 adult patients with chronic sinonasal symptoms showed significantly improved scores [Rhinosinusitis Disability Index (RSDI) and Single-Item Sinus-Symptom Severity Assessment (SIA)] and reduced use of medication, such as antibiotics, for patients with daily hypertonic saline NI (37). Large volume intervention is more efficacious compared to low volume NI (38).

### Pregnancy Rhinitis

Hormonal changes during pregnancy alter AR symptoms and approximately one third of pregnant women observe increased morbidity. There also exists a hormonally—induced rhinitis of pregnancy. However, especially during the first trimester, physicians are reluctant to prescribe drugs, let alone in increased doses. NI is not expected to harm the fetus. Hypertonic saline (3% NaCl) was studied in 45 pregnant women, followed for 6 weeks: the active group had significantly better rhinitis score and less antihistamine use after the first week. Similarly, nasal resistance, albeit similar on week 1, was significantly decreased on weeks 3 and 6. No adverse events were reported and therefore, NI seem to be invaluable for the treatment of rhinitis of pregnant women (39).

### The Common Cold

The role of NI for the therapy of acute upper respiratory infections (URTI- common cold) is not clearly established. Early studies showed no difference in the use of NI for URTI (40, 41), while a large pediatric study of 401 children indicated faster resolution of some nasal symptoms for those that used NI (42). A subsequent Cochrane review acknowledges possible benefits of NI; this, however, is based on small studies with bias risk (43). Similarly, the most recent EPOS guidelines suggest that NI possibly has benefits for relieving symptoms, mainly in children, and could be a therapeutic option (4). Moreover, NI could be used as prophylaxis for prevention of frequent URTI in both children and adults (42, 44).

### Adverse Events

NI does not pose a risk for major adverse events. Epistaxis, nasal and/or aural burning or irritation and middle ear effusions occasionally occur especially with large volume, high pressure, hypertonic solutions. Sodium loading could be problematical in those with concomitant renal or cardiac problems if the solution is swallowed, so advice should be given to spit it out once it reaches the post nasal space. In general NI benefit far outweighs risk.

### Conclusion

NI (especially with hypertonic saline) is a useful add-on to pharmacological treatments and can be used alone in pregnant women, small children and those with mild disease. Further studies are needed to delineate NI use in terms of underlying pathology, volume, tonicity, delivery method, supplementary extracts, or minerals.

## Allergic Rhinitis

**Figure 3:**
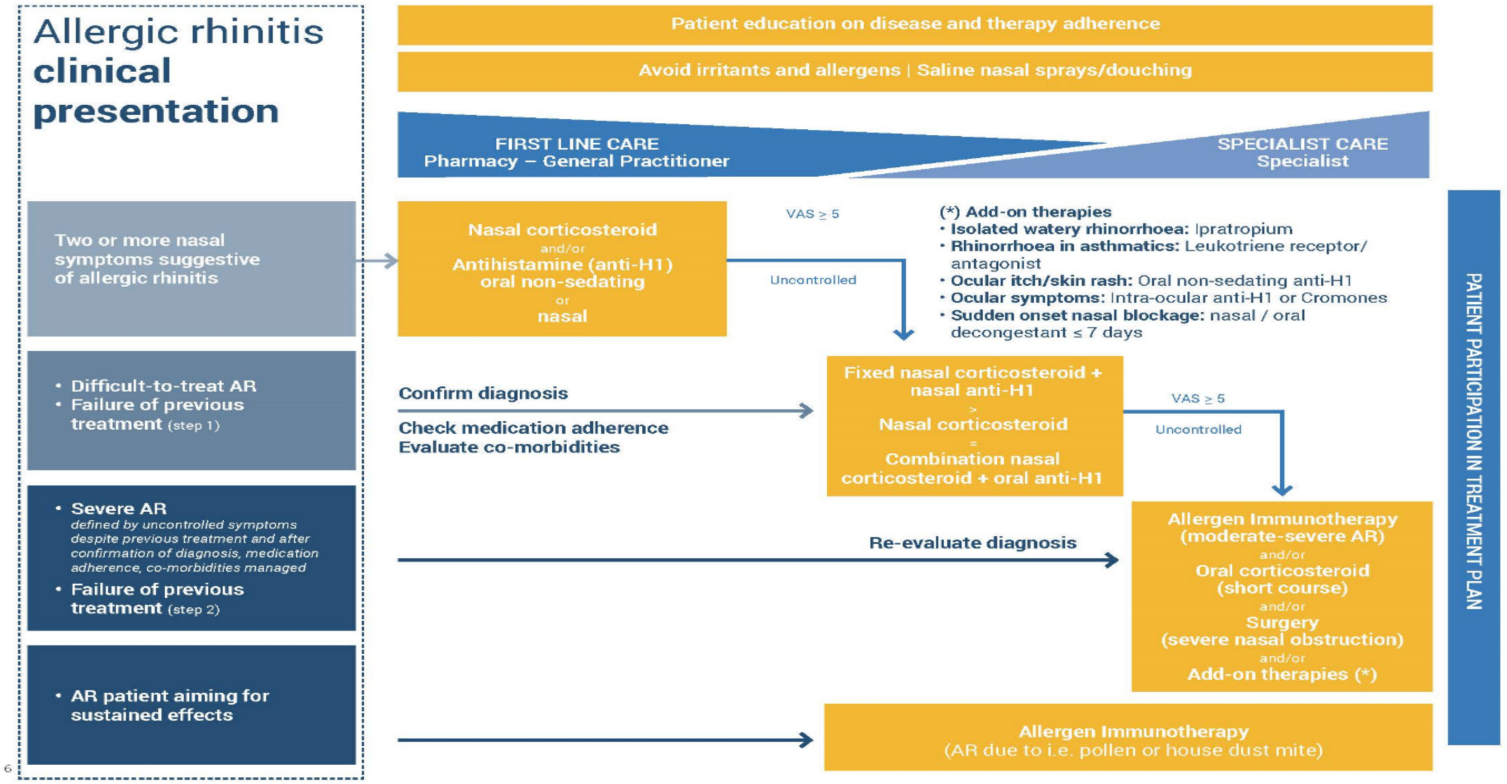
Treatment algorithm for AR as proposed by EUFOREA, taking into account the reality of patient phenotypes and existing international guidelines. EUFOREA treatment algorithm for Allergic Rhinitis (with permission from EUFOREA). The patient should be involved and educated regarding treatment, which starts with allergen and irritant avoidance, plus nasal saline. Further therapies are used as indicated, depending on disease severity and responsiveness to treatment. Failure to control AR should lead to revisiting the diagnosis, the major symptoms, disease extent, and other factors such as patient concordance.

The therapeutic mainstays of Allergic Rhinitis (AR) are antihistamines and corticosteroids, both can be given intranasally, with minimal adverse events, since lower doses can be used. This is particularly important when the severe adverse effects of oral corticosteroid use are considered (45). Figure 3 shows the EUFOREA treatment algorithm for AR.

### Intranasal Antihistamines

Histamine acts in the early phase of allergic responses through H1 receptors (46). Antihistamines are mostly inverse agonists, stabilizing the receptor in an inactive conformation. The H1 receptor is widely distributed throughout the body (besides upper and lower airways in smooth muscle, heart, adrenal medulla, sensory nerves, central nervous system, and others (47) and are G-protein coupled transmembrane receptors that transduce extracellular signals through G proteins to intracellular second messenger systems (48) and may be considered a “cellular switcher,” functioning in equilibrium between two active or inactive conformation states.

Azelastine hydrochloride, levocabastine, and olopatadine hydrochloride are the mostly used intranasal antihistamine (INAH) spray formulations in Europe and the US. The pharmacological profile and clinical efficacy of these drugs have been extensively reviewed elsewhere (49–54). INAH are classified as inverse agonists, as they do not antagonize the binding of histamine, but instead bind to different sites on the receptor (55, 56). Binding of antihistamines to the histamine receptor stabilizes the receptor in the inactive state thereby reducing the intrinsic activity of the receptor in response to histamine (46, 49).

They are classed as second-generation antihistamines with high affinities for the H1 receptor and little affinity for the H2 receptor (57, 58) and typically have a fast onset of action (15 to 30 min) (57, 59, 60) with effects lasting up to 12 h (58, 61). In comparison to oral antihistamines, INAH are more effective at reducing symptoms of itching, rhinorrhoea and sneezing, but less effective at ocular symptoms (62, 63) and have variable effects on nasal congestion (64, 65).

Besides histamine, other mediators released from various immune cells are responsible for amplifying and maintaining inflammation and symptoms. There is some evidence that specific antihistamines including INAH can exert anti-allergic effects beyond inhibiting the action of histamine, including actions on arachidonic acid pathway mediators such as leukotrienes, thromboxanes, inflammatory cells, and mediators (66–70). The mechanisms behind this action have not been fully elucidated but may involve interference with calcium ion channels (50, 54, 71, 72).

The major adverse effect in trials is a bitter taste with azelastine, experienced as severe by a subset (around 10%) of subjects, probably genetic supertasters. It can be mitigated to an extent by correct technique of use as indicated in the manufacturer's advice sheet. The sedating effects of oral azelastine are avoided in the majority of nasal users, since the nasal dose is around one twentieth of the oral one (50).

### Intranasal Corticosteroids (INS)

Topical intranasal corticosteroids (INS) are considered the single most effective treatment for AR and suppress most allergic inflammatory reactions (73). INS have been demonstrated to be more effective for relieving nasal symptoms of AR than oral and intranasal antihistamines (74, 75), especially for nasal congestion (76) and are particularly useful for improving ocular symptoms in AR patients (77, 78). INS also reduce bronchial hyperreactivity (79), as with ocular effects suggesting an effect on neurally—mediated distant symptoms via control of local inflammation and mediator release. Not all INS are equally effective (80).

Beclamethasone was the first steroid to be effectively modified for use in a pressurized INS spray in 1972 (81) and 8 compounds for intranasal application have been approved for AR in Europe and USA including triamcinolone acetonide, budesonide, ciclesonide, mometasone furoate, flunisolide, beclomethasone dipropionate, fluticasone propionate, and fluticasone furoate (73, 82).

Glucocorticosteroids diffuse across cell membranes, therefore lipophilicity is an important property, where they bind to the cytoplasmic glucocorticoid receptor (GR) (primary mechanism) (73, 83). On binding of the GR with the corticosteroid ligand, the heat shock proteins dissociate, allowing the GC-GR complex to translocate into the nucleus or interact with transcription factors in the cytoplasm (84). The anti-inflammatory effects are the result of modifications to gene transcription occurring via transactivation or transrepression. In the transactivation pathway, the activated GC-GR complex migrates to the nucleus where it binds as a dimer to the promotor region of palindromic DNA sequences termed Glucocorticoid Response Elements (GRE) (85). Interaction between the activated GR complex and GRE promotes an increase in the transcription of anti-inflammatory genes and of genes encoding proteins that have inhibitory effects on transcription of inflammatory and immune genes (86). The main anti-inflammatory effects of GCs occur via the suppression of multiple genes that encode inflammatory proteins, a process known as trans-repression (87, 88). INS have been shown to inhibit cytokine production in a range of different cell types. Epithelial generated cytokines act as chemoattractants and recruit effector cells such as eosinophils, basophils, and T cells to the nasal mucosa. Fluticasone propionate or fluticasone furoate significantly reduced levels of GM-CSF, IL-6, and IL-8 in stimulated nasal epithelial cells (89–91). Moreover, fluticasone propionate inhibited the release of IL-4, IL-6, IL-8, and TNF-a at an IC50 of <1 nM (92) in stimulated murine mast cells and to significantly reduce IL-4 and IL-5 levels from stimulated peripheral blood CD4C T cells (93). Different classes of steroid drugs (94) induce a different degree of cytokine inhibition with mometasone furoate being the most potent inhibitor of IL-1, IL-6, and TNF-a production among five different ones (mometasone furoate, hydrocortisone, betamethasone, dexamethasone, and beclomethasone).

Corticosteroids may inhibit the maturation of mast cells via regulating the expression of anti- or pro-apoptotic molecules in mast cell progenitors. Glucocorticoid facilitates apoptosis of eosinophils (95, 96) and reduces the numbers of immune cells, production of Th2 cytokines and chemokines and the release of inflammatory mediators in nasal mucosal samples, mostly they seem to actively target Th2 related cytokines (GM-CSF, IL-6, IL-4, IL-5, IL-10, and IL-13) involved in perpetuating the allergic response, in contrast to Th1 cytokines (IFN-g, IL-2) where no effect of steroid treatment was observed (87).

Again the nasal route involves microgram doses, rather than the milligram ones necessary for oral effectiveness. However, INS vary considerably in their systemic bioavailability and the least bioavailable ones: fluticasone propionate, fluticasone furoate and mometasone furoate should be used in children and when long term use is advisable (15). Unlike topical dermal use, there is no local atrophy from properly—applied INS, probably because of the continual movement of any applied drug by mucociliary clearance. Correct application of the spray onto the lateral wall of the nose, with different directions if two squirts are used, should be taught to every person for whom INS are prescribed or to whom they are sold over the counter. Avoidance of the nasal septum, less well-provided with ciliary action, reduces the chance of epistaxis or the extremely rare complication of septal atrophy (15).

### Combination Therapy

Recently sprays containing both INS and intranasal antihistamine (fluticasone propionate and azelastine hydrochloride; mometasone furoate with olopatadine) have been formulated and tested in AR patients. Both are more effective on symptom reduction compared to either single molecule alone (97). The low pH of the second combination may cause nasal discomfort. Combining INS with intranasal decongestant is slightly more effective than INS alone and does not appear to cause rhinitis medicamentosa (97).

### Intranasal Decongestants

Catecholamines (e.g., phenylephrine) or imidazolines (e.g., oxymetazoline) serve as active agents of intranasal decongestants usually classed as vasoconstrictor sympathomimetic agents (98). Their decongestion effects exert through direct and indirect activation of postsynaptic a1 and a2 adrenergic receptors on smooth muscles lining nasal capacitance vessels). On activation of these receptors, the smooth muscle contraction constricts blood vessels and thus reduces nasal tissue edema (98–100) followed by rapid reduction of nasal congestion (98, 99) without effect on other symptoms of AR [such as nasal itching, rhinorrhea, and sneezing (63, 100, 101). Prolonged or repeated use of decongestants (>3–5 days) may lead rebound swelling and congestion (101, 102) known as rhinitis medicamentosa. Septal atrophy may result from repeated septal application of such sprays and can also occur with the use of intranasal cocaine (103).

### Intranasal Anticholinergics

Intranasal anticholinergic agents (INAA) such as ipratropium bromide can lead to the reduction of rhinorrhoea in AR (104–106) by blocking parasympathetic pathways in the nose that release acetylcholine. Acetylcholine acts on muscarinic receptors on nasal mucus glands to induce hypersecretion (104, 107, 108). Ipratropium bromide is a cholinergic receptor antagonist that blocks the interaction of acetylcholine on muscarinic receptors to inhibit release of watery secretions from mucous glands (104, 107), but has no effect on symptoms of sneezing or nasal congestion or inflammatory responses (104, 109, 110). Side effects include the predictable dry mouth and constipation.

### Intranasal Cromones

Cromones are considered mildly effective in relieving symptoms of nasal itching, rhinorrhoea and sneezing, without affecting nasal congestion (83, 101). Their duration of action is short, requiring frequent dosing (up to four times per day) (101, 111).

Both cromoglicic acid, a derivative of chromone-2-carboxylic acid and nedocromil sodium, a pyranoquinolone, are available as intranasal formulations. The exact mechanism of action of chromones is unknown, although several theories have been postulated. Chromones are thought to exert their anti-inflammatory effects by preventing the release of histamine, tryptase and leukotrienes from mast cells following binding of IgE antibodies to the Fc+RI receptor and crosslinking with allergenic peptides (108, 111, 112). Chromones also have reported effects on eosinophils involved in the allergic response (113), but had no significant effect on basophils (114).

## Non-Allergic Rhinitis (NAR)

When no allergic or other cause is found for nasal symptoms the diagnosis by exclusion of non-allergic rhinitis is made. This exists in two main forms- with and without eosinophilic inflammation. The former can be treated similarly to AR with saline, antihistamines, intranasal corticosteroids, alone or in combination. The latter is neurogenic and may respond to anti-cholinergics, such as ipratropium, or to capsaicin, an extract from chili peppers which reduces overexpression of a cation channel, TRPV1, in the nasal lining. Capsaicin(8-methyl-N-vanillyl-6-nonenamide) is a natural irritant which initially excites neurones, but then has a long refractory period, during which those neurons are unresponsive, not only to capsaicin, but to a variety of stimuli (115). Capsaicin desensitization performed correctly, is safe and effective for reducing NAR symptoms (number needed to treat = 4; 95% confidence interval [CI], 1 to 22) for several months (116). There is insufficient evidence to compare the effectiveness of capsaicin to other topical or systemic medications.

## Acute Rhinosinusitis (ARS)

This is an inflammatory disease affecting the nose and paranasal sinuses with duration up to 12 weeks. Usually initiated by viral infection (common cold) it can be prolonged (post-viral) and, in a few subjects it is complicated by bacterial infection. Nasal saline, decongestants and ipratropium bromide can be used in the common cold; for post viral symptoms INS may help reduce symptoms in adults, but there is a paucity of evidence for any intranasal treatment when bacterial superinfection occurs (117).

## Chronic Rhinosinusitis

Chronic rhinosinusitis (CRS) is a symptomatic inflammatory disease of the nasal and paranasal mucosa lasting more than 12 weeks (118). CRS has polypoid (CRSwNP) and non-polypoid (CRSsNP) subforms (118). Asthma is a prolonged bronchial inflammatory disease with an increased and variable tendency for bronchial contraction (119). Both CRS and asthma are significant health problems; the prevalence of each is ~10% (120), and the prevalence of co-morbid asthma and CRS is ~50% (118). The impact of CRS on the quality of life is significant, analogous to diabetes mellitus (118), and it leads to remarkable costs (121). The main treatment of both CRS and asthma is topically administered corticosteroids and nasal saline douching. Use of corticosteroid locally applied into the maxillary sinus via an indwelling tube was found effective in a group pf HDM sensitive subjects with CRS unresponsive to sinus surgery (122). Corticosteroid- eluting sinus implants reduce polyp size and the need for sinus surgery and are considered an option by EPOS (118). Application using corticosteroids in the nasal douche is now a popular treatment option, but there is no firm evidence for it being more or less effective than when the two are used separately (118).

Topical antifungals and topical antibiotics have been trialed in CRS, without significant benefit, except perhaps in special cases such as tobramycin in cystic fibrosis (118).

However, there is one relatively common CRS subtype in which local nasal therapy, other than saline and corticosteroids, may prove effective.

### Nasal Acetylsalicylic Acid Desensitization in Non-steroidal Anti-inflammatory Drug-Exacerbated Respiratory Disease (N-ERD)

Patients with non-steroidal anti-inflammatory drug (NSAID)—exacerbated respiratory disease (N-ERD) have co-morbid asthma, CRS, and NSAID intolerance often with severe disease forms. They are prone to difficult symptoms and recurrent acute exacerbations despite adequate treatment by local or systemic corticosteroids, nasal saline lavages, antibiotics and sinus surgery. Acetylsalicylic acid (ASA, aspirin) treatment after desensitization (ATAD) may be beneficial. Oral ATAD has been shown to improve the quality of life and sino-nasal symptom scores in patients with N-ERD. However, if ASA is not taken regularly, ATAD is associated with a risk of severe anaphylactoid reactions.

Despite active treatment, some 10–20% of CRS/asthma patients have severe disease, purulent exacerbations and impaired productivity (118, 123, 124). Up to 70% of the uncontrolled cases have Type 2 inflammation, nasal polyps (NP), and/or N-ERD (125–129). The triad of co-morbid CRSwNP, asthma, and N-ERD has previously been called Samter's triad (130). The prevalence of N-ERD is 10–16% in hospital-level CRSwNP patients (131, 132). If endoscopic sinus surgery (ESS) combined with appropriate medical treatment fails, additional therapies including ATAD can be considered to treat N-ERD (118, 133).

Oral ATAD has Level of evidence 1a, although the placebo-controlled studies have had relatively small sample sizes (118). Since ATAD has side- and adverse effects including gastritis, gastrointestinal ulcerations and bleedings, attempts have been made to reduce the risk of side effects. Nasal ATAD (nATAD) tends to have fewer side effects than peroral ATAD both in diagnosis and in therapy (134). The use of nATAD is not suggested (level of evidence 1b-) in the current the European Position Paper on Rhinosinusitis and Nasal Polyps (EPOS 2020), since it lacks sufficient evidence for treatment of CRSwNP patients with N-ERD, but further double- blind studies are recommended (118). Here, we will review the nATAD literature, and communicate our own experience regarding its use.

### Non-steroidal Anti-inflammatory Drug (NSAID) Exacerbated Respiratory Disease (N-ERD)

N-ERD is an inflammatory airway disease usually consisting of a triad of hypersensitivity to NSAIDs, asthma and CRSwNP (130–133). Patients with N-ERD have severe eosinophilic hyperplastic inflammation and fibrotic tissue remodeling in both their paranasal sinuses and lower airways (130, 135, 136). The age of onset for N-ERD is usually around 30 years, it is slightly more common in females (137, 138).

About 9% of asthmatics have N-ERD, the asthma of N-ERD patients tends to be moderate to severe (133). Compared to other asthmatics, N-ERD patients are more likely to need high dose inhaled corticosteroid treatment or steroid bursts (133, 135), and their asthma is more likely to be uncontrolled and to lead to asthma related healthcare visits, hospitalizations, and intubations (135, 137).

CRSwNP treatment of N-ERD patients consists of saline irrigations, nasal steroids, antileukotrienes, oral steroids, oral antimicrobials (139), and endoscopic sinus surgery (ESS) if conservative treatment is not sufficient (118, 135). The need for recurrent sinus surgeries is common in N-ERD patients (140, 141). ATAD (133), and/or biological agents are also considered if other treatments are insufficient (118).

**Figure 4:**
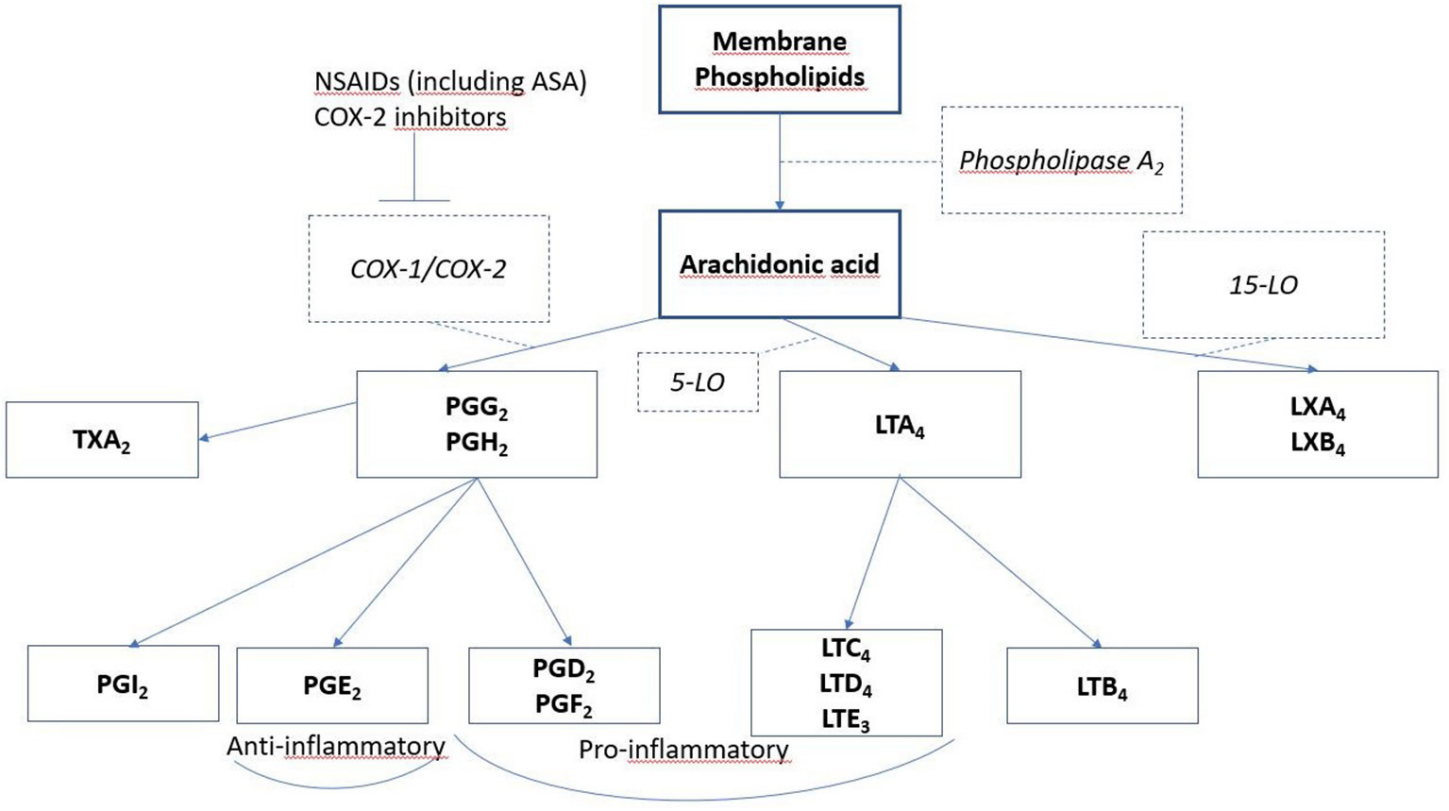
Lipid mediators involved in N-ERD. Arachidonic acid is released from degranulating cells (mast cells and eosinophils) and is metabolized by several routes to form prostaglandins, leukotrienes, and lipoxins. Inhibitors of cyclooxygenase 1, such as aspirin and NSAIDS, block this pathway, reducing bronchoprotective PGE2 and allowing increased pro- inflammatory leukotriene and lipoxin formation.

In N-ERD patients, NSAIDs cause exacerbation of respiratory tract symptoms, provoking nasal congestion, rhinitis and obstruction of the lower airways, usually within 45–60 min of administration, urticaria, dyspepsia, and angioedema can also occur (142). The pathomechanisms behind this are not fully understood; it has been suggested that the hypersensitivity to NSAIDs is not caused by an allergic, immunoglobin E (IgE)—based mechanism, but rather by abnormal metabolism of the lipoxygenase (LO) and cyclooxygenase (COX) pathways (136, 143). Three forms of COX enzyme exist, one of these is COX-1. ASA and its other cross reacting NSAIDs inhibit COX-1, leading to decrease in COX-1 products, including prostaglandins. In N-ERD patients, ingestion of NSAIDs leads to an imbalance in the products of these pathways (143) (Figure 4).

Ideally, N-ERD is diagnosed with a NSAID-challenge test. However, if a patient with confirmed asthma and CRSwNP has had multiple reactions with respiratory symptoms within 2 h after two different NSAID ingestions, this history is sufficient for N-ERD diagnosis (133). In unclear cases, for research purposes, or to evaluate for the provocation dose of ASA in oral desensitization, challenge tests are needed (133). The following contraindications for ASA challenge, ASA desensitization (AD), and ASA treatment after desensitization (ATAD) must be appreciated: prior anaphylactic/anaphylactoid reaction(s) due to NSAIDs, gastrointestinal bleeding, renal failure, uncontrolled asthma (Forced expiratory volume in one second [FEV1] <70% of the predicted value), ongoing respiratory tract infection or asthma exacerbation, current treatment with β-blocker, or pregnancy (133).

The initial use of nasal lysine aspirin (in the USA where this is unavailable, ketorolac is used) for challenge, coupled with sensitive upper airway measurements, means that highly sensitive subjects can be identified at a low dose without causing an asthma exacerbation. A negative nasal challenge necessitates oral challenge with larger doses until 300 mg has been tolerated (144).

### Acetylsalicylic Acid (ASA)

**Figure 5:**
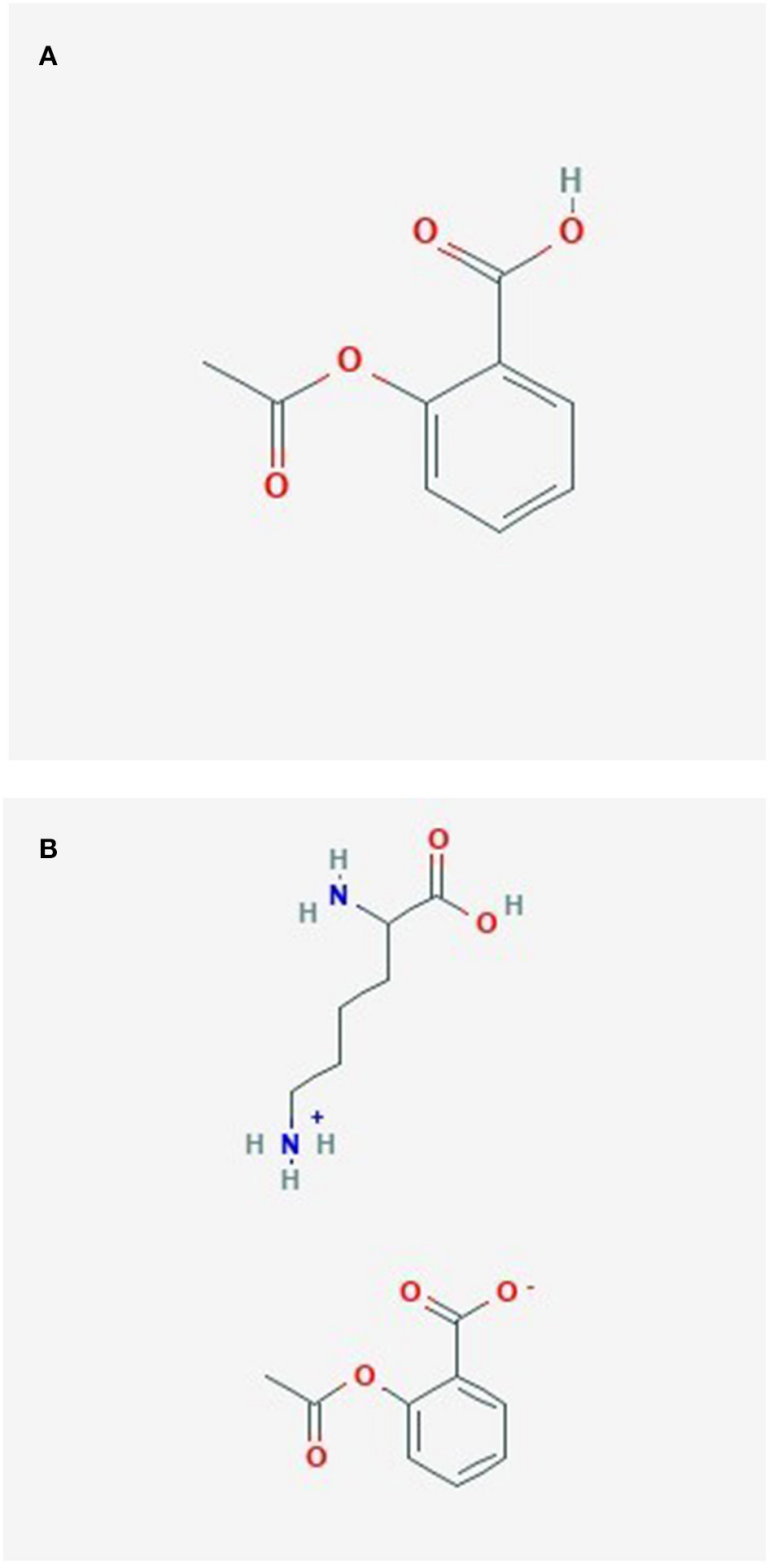
**(A)** Acetylsalicylate. **(B)** Lysine acetylsalicylate.

ASA, also known as aspirin, is a very commonly used drug worldwide (145). It has been used to reduce pain, fever, inflammation, and lately mostly as prevention for cardiovascular diseases, but it may also have other preventive effects (146). The history of acetylsalicylic acid began over 3,500 years ago, when salicylate- containing willow bark was used to treat pain by ancient Sumerians and Egyptians (145). Aspirin was synthesized by Bayer company's chemist Felix Hoffmann in 1897 (145). The molecular formula of ASA is C_9_H_8_O_4_ (146) (Figure 5A).

### Lysine Acetylsalicylate (LAS)

The molecular formula of Lysine acetylsalicylate (Aspirin Lysine salt, Aspirin DL-Lysine, DL-Lysine-acetylsalicylate) is C_15_H_22_N_2_O_6_, its component compounds are DL-Lysine and ASA (147) (Figure 5B). Lysine acetylsalicylate (LAS) is soluble, it is the only genuinely soluble aspirin preparation (148) and was developed for intravenous administration to treat pain (148, 149).

### ASA Treatment After Desensitization (ATAD) in N-ERD Patients

Due to the severity of symptoms in N-ERD, there has been an interest to improve its treatment, one of these developments is ATAD. Since the ASA intolerance of N-ERD patients is not due to IgE-mediated allergy, ATAD is not comparable to allergen desensitization in IgE confirmed allergic diseases. The aim of ATAD is to reduce polyp growth and decrease (upper) airway symptoms. ATAD is considered in N-ERD patients with insufficient response to pharmacological treatment, high recurrence of NPs leading to recurrent surgeries, insufficient control of asthma symptoms with standard medications, need to reduce corticosteroid dose, or in patients who need ASA or NSAID treatment (133, 150).

Our real-world follow-up study showed high discontinuation rates of peroral ATAD with a lack of effect on revision sinus surgery rates, prescribed antibiotics and oral corticosteroid courses (151). The latest EPOS 2020, however, concludes that peroral ATAD improves the quality of life and total nasal symptom scores in patients with N-ERD (118, 147, 152–155).

AD can be performed in an outpatient setting as an extension of ASA challenge, with ATAD continuing straight after the challenge by gradually increasing ASA doses (133, 144, 150). ATAD is usually performed with peroral ASA, with the effective dose varying between 300 and 1,300 mg daily (133, 147, 155–161). Intranasal ASA has been used in both ASA challenge and ATAD (133, 134). There is some evidence, that the surgical removal of NPs, or ESS may be beneficial prior to ATAD (133, 136, 144, 158–162).

### Nasal Lysine Aspirin Challenge in N-ERD Patients

Although thus far not a routine part of clinical diagnostics, nasal challenge test with Lysine aspirin (LAS) was introduced for N-ERD assessment in the 1990s (163). The LAS doses, duration of observation period, and criteria for positivity have varied in different studies. One study group performed nasal ASA challenge tests (ASA-NCT) for 51 patients with N-ERD, confirmed by oral ASA challenge (163). The study did not report systemic reactions, including bronchospasm. They concluded that ASA-NCT is highly specific (95.7%) and sensitive (86.7%), that the nasal test is simple, safe, and quick for N-ERD diagnostics, but that negative results do not exclude possible ASA intolerance (163). Another study group performed ASA-NCT with relatively little side effects and showed positive result in 100 of 131 patients with severe CRSwNP and asthma (164). This study concluded that provided patients are carefully chosen and monitored, ASA-NCT is suitable for day-case practice (164).

### Nasal Lysine Aspirin Treatment After Desensitization in N-ERD Patients

Nasal Lysine Aspirin treatment after desensitization (nATAD) has been used to treat N-ERD patients, but it has not been in wide clinical use. In the one existing double blind placebo controlled trial by Parikh and Scadding, 22 subjects with ASA sensitive nasal polyposis were enrolled, they were randomized to receive either 16 mg of topical LAS or placebo every 48 h for 6 months before cross-over. Only 11 study subjects completed the study, and no clinical benefit could be demonstrated (165) (Table 1). However, a reduction in the characteristically elevated levels of cysLT1 receptors was seen (169) and confirmed in a further study which also showed that this phenomenon did not occur in aspirin tolerant subjects (170).

**Table 1:** Trials of intranasal lysine aspirin in nasal polyposis.

**References**	**Study method**	**Study participants**	**Dose**	**Outcome measures**	**Study results**
Patriarca et al. (166)	Prospective, non-randomized controls	20 patients with N-ERD and CRSwNP/43 patients with CRSwNP 191 control patients	2 mg (ASA equivalent) per week	NP relapse	NP relapse rate decreased in LAS group
Nucera et al. (167)	Prospective, non-randomized controls	(1) 28 (N-ERD+CRSwNP)/ out of 76 patients. (2) 14 (N-ER+CRSwNP)/out of 49 patients Control group 191 CRSwNP patients	4 mg (ASA equivalent) 6 times per week	Recurrence of NP (in CT and clinical control)	Recurrence of NPs reduced in LAS group
Parikh and Scadding (165)	Double blind placebo controlled cross-over trial	22 ASA intolerant patients (of these 19 had CRSwNP), 11 completed the study	16 mg (ASA equivalent) every 48 h for 6 months before cross-over	Nasal and pulmonary symptom scores ARM PEF rate PNIF	No significant differences between the groups But cysLT1 receptors reduced
Ogata et al. (168)	Prospective, open n of 1 study	13	54 mg LAS [ASA equivalent 37.8 mg (53)] per day	NP volume NIPF, nNO, eNO, PEFR	NP volume reduced, NIPF, and nNO improved
Howe et al. (134)	Audit	105 AERD + LAS treatment/out of 121 patients with AERD	75–100 mg ASA equivalent per day	Subjective symptom evaluation + VAS PNIF Exhaled + nasal NO Olfaction Spirometry Asthma questionnaire	Symptom improvement Reduced airway inflammation Improvement of olfaction Improvement of asthma outcomes

*Nasal Lysine aspirin (LAS) treatment, N-ERD (Non-steroidal anti-inflammatory drug exacerbated respiratory disease) patients with CRSwNP (chronic rhinosinusitis with nasal polyposis). N-ERD, Non-steroidal anti-inflammatory drug exacerbated respiratory disease; AERD, aspirin exacerbated respiratory disease; LAS, Lysine aspirin; CRSwNP, chronic rhinosinusitis with nasal polyps; NP, nasal polyps; ASA, aspirin; CT, computed tomography; ARM, acoustic rhinometry; PEF, peak expiratory flow; PNIF, Nasal inspiratory peak flow; VAS, visual analog scale; NO, nitric oxide*.

Prospective, non-randomized studies have shown clinical benefits in LAS treated NP patients (166–168, 171) (Table 1). In an n of 1 study 13 N-ERD subjects who were uncontrolled on standard therapy were studied for 3 months and then for a further 3 months with the addition of nasal lysine aspirin, gradually increased to 54 mg daily (168). Significant improvement was seen in nasal inspiratory peak flow rate, *p* = 0.014) and nasal nitric oxide levels rose significantly (in both sides, *p* = 0.028), suggesting opening up of the nasal airway and sinus orifices. Exhaled nitric oxide and peak expiratory flow did not change. Compared with the preceding 3 months, adding intranasal lysine-aspirin had an effect on decreasing nasal polyp volume (right side, *p* = 0.031; left side, *p* = 0.016) (168).

Howe et al. (134) performed a non-controlled audit study including 105 N-ERD patients with intranasal LAS in gradually increasing doses following positive LAS challenge. Symptoms improved/stabilized in over 70% subjects at 3 and 12 months, and nasal inspiratory peak flow, olfaction, exhaled and nasal nitric oxide levels were also improved significantly. Asthma outcomes, including use of oral corticosteroids, exacerbations and emergency visits were all reduced in 22 subjects taking lysine aspirin over a year, compared to 20 challenge- positive subjects who ceased using it. Gastrointestinal side effects occurred in 3.8%, which is lower than those reported for oral ASA therapy (145). LAS has also been studied in treatment of patients with CRSwNP but no ASA intolerance. A prospective study involving 20 patients with CRSwNP but no ASA intolerance, receiving 2,000 μg LAS in one nostril and saline in one nostril, showed that polyp recurrence tended to be milder on the LAS treated side. However, a double blind, placebo- controlled trial was negative in aspirin- tolerant nasal polyp patients (172).

### Finnish Experience With Nasal Lysine Aspirin Desensitization in N-ERD Patients

Seven nasal aspirin challenge- positive subjects [at 10 mg (*n* = 2) and 20 mg, *n* = 5] were given nasal ASA-desensitization (nAD) according to an earlier published method (134, 173), at the Department of Otorhinolaryngology—Head and Neck Surgery of Helsinki University Hospital.

Six of the seven patients discontinued the nAD (mean duration of the desensitization 19.2 days, range 7–39 days). The known reasons for the discontinuation were severe abdominal pain in three in one accompanied by an asthma exacerbation, and exacerbation of nasal blockage in two. Fortunately, the side effects were transient when nAD was discontinued.

Only one patient continued nAD. Although the dose was low (50 mg) the patient felt symptom relief. Uveitis developed 2 months after onset of nAD, however, according to the ophthalmologist it was not caused by the nAD.

It is possible that the Finnish population does not tolerate ATAD as well as other populations (151), certainly the high reports of gastrointestinal symptoms are concerning. However, it is necessary to warn patients that the desensitization process takes time and that usually they will be worse before their condition improves. Gradual updosing is necessary, with reduction of the dose when adverse events become problematical, as in allergen immunotherapy, although the mechanism of effect of ATAD appears more likely to relate to exhaustion of mediator- bearing cells and to receptor downregulation.

### Conclusions

Nasal acetylsalicylic acid treatment after desensitization is a treatment option for N-ERD. The evidence of its benefits for N-ERD patients is not yet convincing, further randomized double-blind placebo-controlled studies are needed. According to the literature, nasal acetylsalicylic acid treatment after desensitization causes fewer side effects than oral ATAD, our own limited experience, however, contradicts this.

## Intranasal Drugs for Diseases Outside the Nose

While the intranasal administration of drugs for the treatment of nasal diseases is well-established, intranasal drug delivery is increasingly recognized as being a useful and reliable alternative to oral and parenteral application of drugs for systemic diseases and the nasal mucosa has seriously emerged as a therapeutically viable route for systemic drug delivery. In particular nasal delivery seems to be able to circumvent the blood-brain barrier allowing direct drug delivery in the biophase of central nervous system-active compounds. Also, pharmacologically active compounds with poor stability in gastrointestinal fluids, poor intestinal absorption or unfavorable gastrointestinal and hepatic pre-systemic metabolism are of interest.

Peptide drugs (hormone replacement) treatments in different diseases appear to provide good indications under these circumstances. Different peptide hormones are available as nasal sprays, e.g., authorized products exist for estradiol steroid substitution of estradiol (Aerodiol®) (174, 175) and Gonadorelin hormone for undescended testicle (Kryptocur®) (176).

For the treatment of diabetes insipidus, the peptide analog desmopressin is available for both, nasal and oral administration with a given bioavailability of the commercial tablet of 0.1% and of 3–5% for the nasal spray. It can also be used for nocturnal enuresis in children and in multiple sclerosis. Recently the desmopressin spray was withdrawn from use for mild hemophilia and von Willebrand's disease because of higher than specified dosage^1^. Too much desmopressin can cause sodium levels in the blood to drop sufficiently to result in seizures, coma, and death.

Syntocinon nasal spray containing oxytocin is used to increase duration and strength of contractions during labor and has been investigated for some psychiatric conditions such as anorexia nervosa, autism, anxiety disorders, schizophrenia and alcohol deprivation (177).

Gonadotropin-Releasing-Hormone (GnRH) analogs such as Nafarelin (Synarel®) and busurelin are used for the treatment of endometriosis, precocious puberty, anovulatory infertility, hypogonadotropic, and cryptorchidism (178).

Further authorized products exist for nicotine withdrawal for smoking cessation (Nicotrol NS®) (179).

## Future Uses of the Intranasal Route

Anti- viral molecules are under investigation for the reduction of COVID-19 transmission^2^.

Since there is some evidence for an intranasal, virally—mediated etiology for some neurodegenerative conditions (Parkinson's and Alzheimer's diseases) it may eventually be possible to prevent these by prophylactic use of a non-toxic intranasal antiviral.

Intranasal adrenalin is under trial for urgent anaphylaxis therapy. It will be necessary to block the nasal mucosa from giving a vasoconstrictive response to the applied adrenalin.

The united airways concept, wherein the nose and lower airways react as one unit to stimuli (180, 181) has led to some attempts to treat both areas via the nose, rather than using both nasal spray and inhaler. As yet this sensible concept has not proved successful.

Immunologically active intranasal preparations will be considered in Part 2.

## Conclusion

The nose provides a useful route for therapy of airways diseases and also for other conditions such as CNS and endocrine disorders. Its accessibility, simplicity of use, good blood flow and protective epithelium should allow it to be investigated further as an alternative to systemic administration.

## Author Contributions

CC and NB contributed the section on nasal structure and function and Figures 1, 2. DM and NP contributed the saline section. LK contributed intranasal drugs. AL-H, MH, and ST-S contributed the section on lysine aspirin. GS conceived the idea of the paper, edited the contributions, and wrote the Abstract, Introduction, and Conclusion. All authors contributed to the article and approved the submitted version.

## Conflict of Interest

AL-H reports receiving funding from Orion Research Foundation, outside of this submitted work. GS reports fees and consultancies from ALK, Mylan, Bayer, GSK outside the submitted work. ST-S reports a grant of GSK and consultancies for ERT, Novartis, Sanofi Pharma and Roche outside the submitted work. LK reports grants and personal fees from Allergopharma, grants and personal fees from MEDA/Mylan, personal fees from HAL Allergie, grants from ALK Abelló, grants and personal fees from LETI Pharma, grants from Stallergenes, grants from Quintiles, grants and personal fees from Sanofi, grants from ASIT biotech, grants from Lofarma, personal fees from Allergy Therapeut., grants from AstraZeneca, grants from GSK, grants from Inmunotk, personal fees from Cassella med, outside the submitted work; and Membership of AeDA, DGHNO, Deutsche Akademie für Allergologie und klinische Immunologie, HNO-BV, GPA, EAACI. The remaining authors declare that the research was conducted in the absence of any commercial or financial relationships that could be construed as a potential conflict of interest.

## Footnotes

^1^Available online at: https://hemophilianewstoday.com/2020/10/05/recall-on-ferrings-intranasal-desmopressin-therapies-to-affect-availability-into-2021/

^2^Available online at: https://www.itv.com/news/central/2020-11-16/new-face-mask-that-kills-coronavirus-could-be-available-by-december-says-nottingham-scientist-nottingham-trent-university-dr-gareth-cave?utm_source=upday&utm_medium=referral
